# Tomographic assessment of thoracic fungal diseases: a pattern and signs approach

**DOI:** 10.1590/0100-3984.2017.0223

**Published:** 2018

**Authors:** Pedro Paulo Teixeira e Silva Torres, Marcelo Fouad Rabahi, Maria Auxiliadora Carmo Moreira, Pablo Rydz Pinheiro Santana, Antônio Carlos Portugal Gomes, Edson Marchiori

**Affiliations:** 1 Multimagem Diagnósticos, Goiânia, GO, Brazil.; 2 Department of Internal Medicine, School of Medicine, Universidade Federal de Goiás (UFG), Goiânia, GO, Brazil.; 3 Med Imagem/Hospital Beneficência Portuguesa and Grupo Fleury, São Paulo, SP, Brazil.; 4 Med Imagem/Hospital Beneficência Portuguesa, São Paulo, SP, Brazil.; 5 Department of Radiology, Universidade Federal do Rio de Janeiro (UFRJ), Rio de Janeiro, RJ, Brazil.

**Keywords:** Invasive fungal infections, Tomography, X-ray computed, Diagnostic imaging, Infecções fúngicas invasivas, Tomografia computadorizada, Diagnóstico por imagem

## Abstract

Pulmonary fungal infections, which can be opportunistic or endemic, lead to considerable morbidity and mortality. Such infections have multiple clinical presentations and imaging patterns, overlapping with those of various other diseases, complicating the diagnostic approach. Given the immensity of Brazil, knowledge of the epidemiological context of pulmonary fungal infections in the various regions of the country is paramount when considering their differential diagnoses. In addition, defining the patient immunological status will facilitate the identification of opportunistic infections, such as those occurring in patients with AIDS or febrile neutropenia. Histoplasmosis, coccidioidomycosis, and paracoccidioidomycosis usually affect immunocompetent patients, whereas aspergillosis, candidiasis, cryptococcosis, and pneumocystosis tend to affect those who are immunocompromised. Ground-glass opacities, nodules, consolidations, a miliary pattern, cavitary lesions, the halo sign/reversed halo sign, and bronchiectasis are typical imaging patterns in the lungs and will be described individually, as will less common lesions such as pleural effusion, mediastinal lesions, pleural effusion, and chest wall involvement. Interpreting such tomographic patterns/signs on computed tomography scans together with the patient immunological status and epidemiological context can facilitate the differential diagnosis by narrowing the options.

## Abstract (pt)

Pneumopatias fúngicas proporcionam considerável morbidade e mortalidade, podendo ser oportunistas ou endêmicas. De maneira geral, as apresentações clínicas e padrões de imagem são múltiplos e superponíveis a várias doenças, dificultando a abordagem diagnóstica. Tendo em conta a amplitude do território nacional, o conhecimento da realidade epidemiológica dessas doenças em cada região é fundamental para a consideração delas no diagnóstico diferencial. A definição do estado imunológico irá, ainda, definir a possibilidade de doenças fúngicas oportunistas, por exemplo, na síndrome da imunodeficiência adquirida ou em situações de neutropenia febril. Em geral, histoplasmose, coccidioidomicose e paracoccidioidomicose comprometem indivíduos imunocompetentes, e aspergilose, candidíase, criptococose e pneumocistose comprometem indivíduos imunodeprimidos. Vidro fosco, nódulos, consolidações, micronódulos de disseminação miliar, lesões escavadas, sinal do halo/halo invertido e bronquiectasias são padrões tomográficos frequentes no acometimento pulmonar e serão abordados individualmente, além de apresentações menos frequentes, como lesões mediastinais, derrame pleural e acometimento da parede torácica. A interpretação desses padrões/sinais tomográficos básicos associados a dados epidemiológicos e estado imunológico do paciente pode ser útil, contribuindo para o estreitamento das opções diagnósticas.

## INTRODUCTION

Pulmonary fungal infections, which can be opportunistic or endemic, are associated with considerable morbidity and mortality. In recent decades, there has been a significant increase in the incidence of such infections, due to medical treatments (immunosuppressive therapy, transplantation, and the use or abuse of antibiotics), as well as to the rising incidence of leukemia, lymphoma, and AIDS, together with the improved accuracy of diagnostic techniques^(1)^. In general, histoplasmosis, coccidioidomycosis, and paracoccidioidomycosis affect immunocompetent individuals, whereas aspergillosis, candidiasis, cryptococcosis, and pneumocystosis affect immunocompromised individuals. There are multiple clinical scenarios, with variable and overlapping imaging patterns. Therefore, it is essential that attending physicians be familiar with the epidemiological status of the fungi in their region, the immunological status of the patient, and the imaging patterns of each entity^(2-4)^.

In this study, we will describe the pulmonary fungal infections that are most common in Brazil, discussing the main tomographic patterns observed in the pulmonary and mediastinal compartments.

## GEOGRAPHIC DISTRIBUTION OF ENDEMIC PULMONARY FUNGAL INFECTIONS IN BRAZIL

Data on the distribution of fungal infections in Brazil are scarce, and knowledge of endemic areas is based on reports of clinical cases and intradermal investigations, as are the prevalence, incidence, and morbidity data for some conditions^(5)^. Endemic fungal infections with pulmonary involvement are not on the national mandatory reporting list^(6)^. It is assumed that the epidemiology of these infections is changing, partly due to global climate changes, new agricultural practices (widespread mechanization and the use of fungicides), human migration, adventure tourism, and other causes^(7)^.

Paracoccidioidomycosis is considered an endemic disease, approximately 80% of all cases occurring in Brazil, mainly in the states of São Paulo, Paraná, Rio Grande do Sul, Goiás, Rio de Janeiro, and Rondônia^(8,9)^. Cases have also been reported in areas inhabited more recently and undergoing deforestation, such as in parts of the Brazilian Amazon rain forest, including those in the states of Amazonas, Maranhão, Tocantins, Pará, Mato Grosso, Rondônia, Acre, and Amazonas^(5,10)^.

The number of reported cases of coccidioidomycosis is highest in the states in northeastern Brazil, and the last endemic area defined for this mycosis in the Americas comprises the states of Piauí, Ceará, Maranhão, and Bahia^(9,11,12)^. There have been 26 reported microepidemics of histoplasmosis, in seven Brazilian states (Rio de Janeiro, Rio Grande do Sul, São Paulo, Minas Gerais, Paraíba, Amazonas, and Bahia) and the Federal District of Brasília, with isolation of the fungus in Rio de Janeiro, Rio Grande do Sul, São Paulo, Paraíba, and the Federal District of Brasília^(13)^.

## PULMONARY FUNGAL INFECTIONS IN IMMUNOCOMPROMISED INDIVIDUALS

Fungal infections are among the most serious infections in immunocompromised individuals, and pulmonary involvement remains the most common documented form of invasive tissue infection in immunocompromised hosts^(14)^. In general, some fungal infections, including pneumocystosis, cryptococcosis, and aspergillosis, have a predilection for immunocompromised individuals^(3,4,15)^. Other agents implicated are fungi of the genera *Mucor*, *Fusarium*, *Rhizopus*, *Petriellidium*, *Cryptococcus*, *Histoplasma*, *Coccidioides*, and *Candida*. Although infections with *Aspergillus* sp. are still the most common fungal infections among immunocompromised individuals in the United States and Europe, the prevalence of HIV infection has made infections with fungi of the genera *Cryptococcus* and *Pneumocystis* the most common fungal infections among such individuals in other parts of the world^(16)^.

Populations at high risk for pulmonary fungal infections include individuals with solid or hematological malignancies, those undergoing organ or bone marrow transplantation, and HIV infected patients. Others who are at intermediate risk include patients on chronic corticosteroid or immunosuppressive therapy, those with chronic kidney disease, those with chronic obstructive pulmonary disease (COPD), and those with liver cirrhosis^(14,16)^. In this subgroup of individuals, an aggressive etiological investigation protocol is necessary, because a diagnostic delay increases mortality, and early use of computed tomography in diagnostic protocols is recommended^(14)^.

## TOMOGRAPHIC PATTERNS OF THORACIC INVOLVEMENT

### Ground-glass opacities

**Figure 1:**
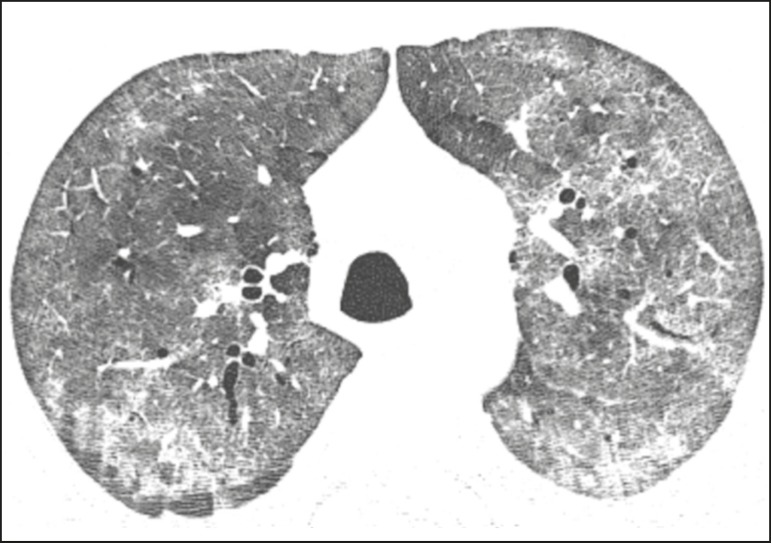
HIV-positive patient with pneumocystosis. High-resolution computed tomography of the upper lung lobes, showing diffuse ground-glass opacities in the pulmonary parenchyma and sparse small foci of consolidation.

In patients with *Pneumocystis jirovecii* pneumonia or pneumocystosis, the most characteristic finding on high-resolution computed tomography scans is that of ground-glass opacities, reflecting intra-alveolar accumulation of fibrin, debris, and microorganisms. On high-resolution computed tomography, the aspect is that of diffuse lung disease, with ground-glass opacities that tend to appear predominantly in the upper lobes and rarely on the lung periphery^(17)^. Superimposed on ground-glass opacities, reticular opacities can be observed, characterizing the crazy-paving pattern (Figure 1). In some cases, discrete, bilateral, uniform ground-glass opacity may be difficult to detect, and it can be helpful comparing the discrepancy between the attenuation of the pulmonary parenchyma and that of the bronchial air content, described as the “dark bronchus” sign^(18)^. Ground-glass opacity can also be accompanied by cysts or consolidations, the latter being more common in non-HIV-infected individuals^(17)^.

Ground-glass opacities, typically with a sparse distribution, are the most common finding in patients with untreated paracoccidioidomycosis^(19)^. They reflect intralobular interstitial thickening due to inflammation, with or without air space filling, or due to intralobular fibrosis^(20)^. In the chronic manifestation of the disease or after treatment, the changes evolve to fibrosis, with the appearance of reticular opacities characterized by thickening of the peribronchovascular bundle, emphysema adjacent to areas of parenchymal scarring, traction bronchiectasis, parenchymal bands, and architectural distortion^(20,21)^.

### Nodules

Nodules are common findings in pulmonary mycoses, including cryptococcosis, paracoccidioidomycosis, and coccidioidomycosis, as well as fungal infections with angioinvasive manifestations, such as *Aspergillus* sp. infections, mucormycosis, and candidiasis^(4,20,22,23)^.

**Figure 2:**
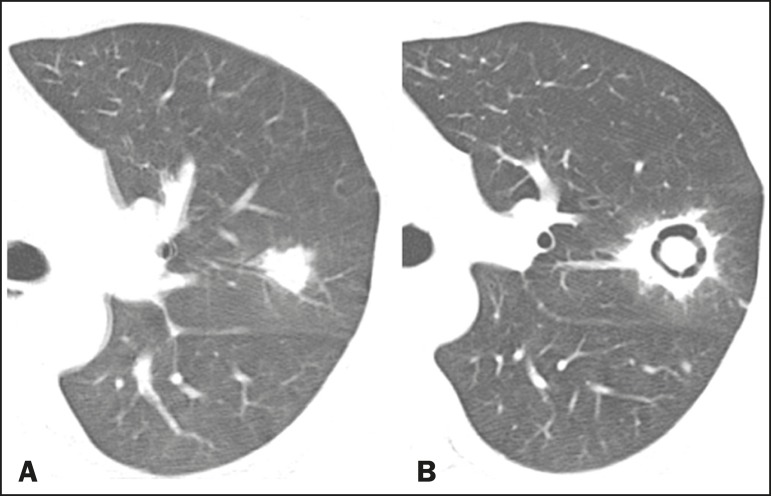
Patient with acute lymphoid leukemia, febrile neutropenia, and angioinvasive fungal infection. A: Axial tomography scan of the chest showing an irregular nodule with a discrete ground-glass halo (halo sign). B: After antifungal therapy had been started, there was cavitation of the nodule, with an intracavitary nodule (air crescent sign).

**Figure 3:**
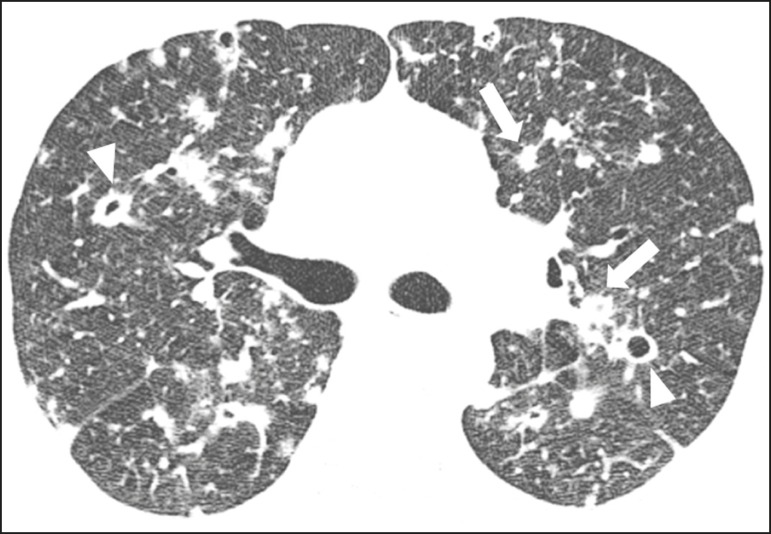
Patient with untreated paracoccidioidomycosis. Axial high-resolution computed tomography scan of the chest, showing multiple sparse irregular nodules (arrows), some cavitary (arrowheads).

Multiple nodules that are predominantly peripheral, especially when accompanied by a ground-glass halo, are typical of the angioinvasive manifestation of aspergillosis in patients with febrile neutropenia^(23)^. A similar aspect can be observed in angioinvasive infections with other fungi, such as those of the order Mucorales and *Candida* sp.^(23)^. In the evolution of the disease, the nodules progress to central necrosis and cavitation, with the appearance of air within the lesions, known as the “air crescent” sign, morphological characteristics typically observed two to three weeks after the start of treatment (Figure 2), coinciding with the resolution of neutropenia^(23-25)^. Mobile nodules within the lung cavitation are also characteristic of the saprophytic presentation of aspergillosis^(23)^. Nodules with or without cavitation, as well as centrilobular nodular opacities, which are common patterns in untreated paracoccidioidomycosis, are typically seen in the peripheral and posterior lung fields, with a slight predominance in the middle lung fields^(19)^, as shown in Figure 3.

**Figure 4:**
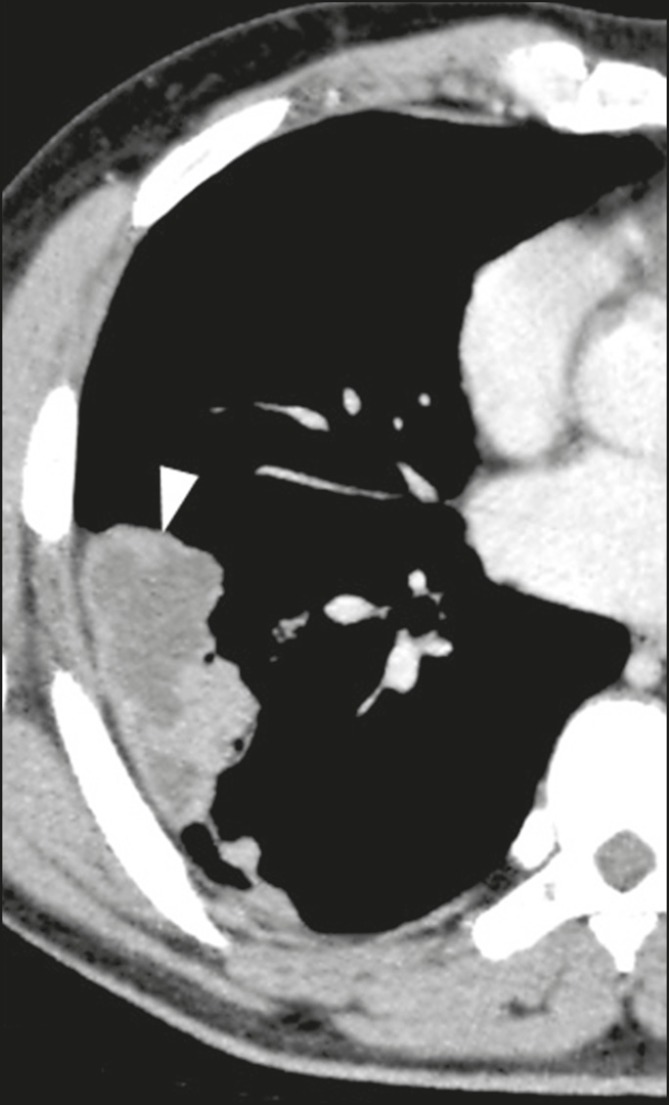
Patient with cryptococcosis. Contrast-enhanced axial tomography scan of the chest, with a mediastinal window setting, showing lobular peripheral consolidation with a central necrosis component (arrowhead).

Pulmonary nodules and masses predominate in cryptococcosis, usually with peripheral distribution (Figure 4). Although nodule cavitation can be observed in immunocompetent and immunocompromised patients, it is reported to be more common in the latter group^(3,22)^.

The typical tomographic presentation of the acute form of coccidioidomycosis consists of irregular nodules of varying dimensions with a tendency toward confluence and cavitation, a pattern that is often seen in individuals from semi-arid regions in northeastern Brazil^(26)^. There is an association between the disease and the practice of armadillo hunting, and the disseminated aspect seen on imaging could be due to massive aspiration of fungi from contaminated soil during the process of extracting the animal from its hole^(26)^.

It is of note that the nodular presentation of fungal infections can be confused with that of neoplastic diseases, especially when the nodules are irregular^(27)^.

### The halo and reversed halo signs

The halo sign refers to ground-glass attenuation surrounding a nodule and was first described in angioinvasive aspergillosis affecting immunocompromised individuals, histopathologically corresponding to an area of infarction surrounded by alveolar hemorrhage^(24,28)^. However, the halo sign, in isolation, is nonspecific and can be observed in a variety of infectious (fungal, bacterial, viral, and parasitic), inflammatory, and neoplastic diseases^(28)^. Among fungal infections, the sign has been observed not only in aspergillosis but also in mucormycosis, candidiasis, coccidioidomycosis, and cryptococcosis^(28)^.

The halo sign is of particular importance in immunocompromised individuals, in whom its specificity is greater for the angioinvasive presentation of some fungal infections, especially aspergillosis. Its occurrence is usually transient, being more common in the early stages of the disease^(29)^. For the differential diagnosis, it should be noted that thickness of the ground-glass halo is often greater in infectious conditions^(30)^.

**Figure 5:**
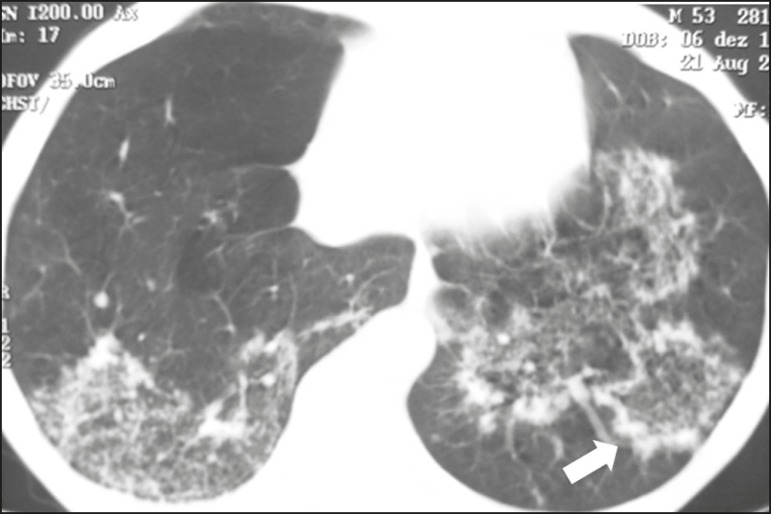
Patient with paracoccidioidomycosis. Axial tomography of the chest, with a lung window setting, showing numerous sparse groups of micronodules and a lesion with the reversed halo sign in the left lower lobe (arrow).

The reversed halo sign refers to a focal ground-glass opacity surrounded by a full or partial ring of consolidation^(24)^. It can be observed in a variety of infectious and noninfectious diseases; among fungal infections, it is more common in paracoccidioidomycosis and angioinvasive fungal infections such as aspergillosis, zygomycosis and fusariosis^(31,32)^. In an attempt to narrow the differential diagnosis of diseases presenting with the reversed halo sign, findings of internal reticulation, peripheral halo thickness > 1.0 cm and pleural effusion favor the diagnosis of an angioinvasive fungal infection over that of organizing pneumonia, within the appropriate clinical context of immunosuppression^(33,34)^. A variation of the reversed halo, with nodular margins (Figure 5), can be seen in paracoccidioidomycosis, as well as in other granulomatous diseases such as tuberculosis^(31,33,35)^.

### Consolidations

Airspace consolidations may be seen in various fungal infections^(2-4,20,22,23)^. After the nodular pattern, it is the second most common finding in cryptococcosis, with a predominantly peripheral distribution^(22)^. Airspace consolidation can also be seen in infections with other endemic fungi, such as paracoccidioidomycosis (referred to as the pneumonic form), histoplasmosis, and acute coccidioidomycosis^(4,26)^. Among immunocompromised individuals, angioinvasive fungal infection (aspergillosis, mucormycosis, or candidiasis) can lead to consolidations, usually with a peripheral distribution, as well as with a ground-glass halo caused by alveolar hemorrhage^(4,23)^. Consolidations are described as part of the semi-invasive, airway-invasive, and bronchopulmonary allergic manifestations of aspergillosis^(23)^. Advanced stages of pneumocystosis can also include consolidation, and its occurrence is more common in non-HIV-infected patients, in whom it tends to develop faster, reflecting the lung damage caused by the host immune response^(17)^.

### Miliary pattern

**Figure 6:**
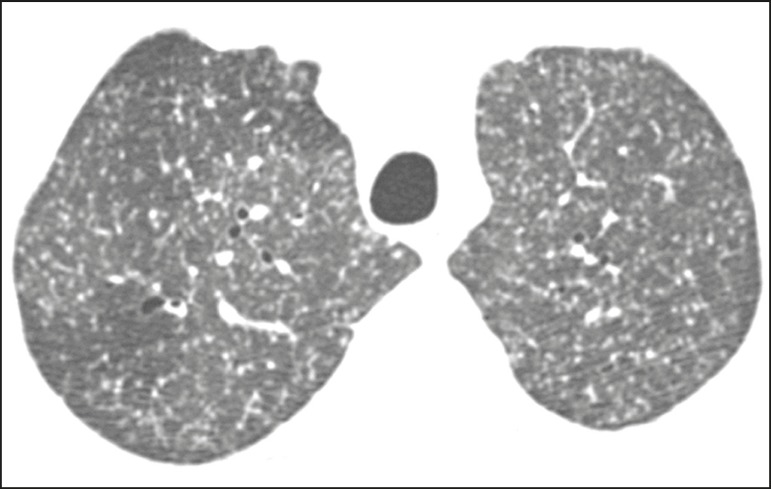
Patient with miliary histoplasmosis. Axial tomography of the chest, with a lung window setting, showing micronodules with a random distribution.

Micronodules with a random pattern of dissemination (miliary pattern) constitute a common presentation in various pulmonary mycoses, being described most frequently in histoplasmosis (Figure 6), paracoccidioidomycosis, coccidioidomycosis, and candidiasis, the last being more common in immunocompromised patients^(4,20)^. The differential diagnoses of a miliary pattern include miliary metastases and the hematogenous dissemination of tuberculosis.

### Cavitations

Cavitary lesions can be observed in various fungal infections, such as angioinvasive disease (aspergillosis, mucormycosis, and candidiasis), as well as in the semi-invasive (necrotizing) and saprophytic manifestations of aspergillosis, histoplasmosis, paracoccidioidomycosis, cryptococcosis, and coccidioidomycosis^(36)^.

**Figure 7:**
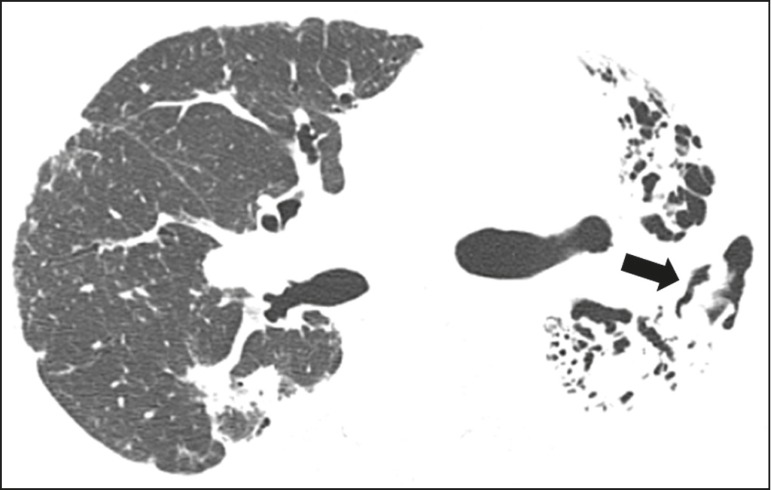
Patient with a history of acute lymphoid leukemia and chronic necrotizing aspergillosis. Axial high-resolution computed tomography of the chest, showing chronic consolidations in the left lung lobes, accompanied by bronchiectasis, with a prominent, filled cavitary lesion in the left lower lobe (arrow).

Semi-invasive (necrotizing) aspergillosis is the chronic granulomatous manifestation of aspergillosis, presenting clinically as a productive cough, fever, and hemoptysis lasting for several months^(4)^. It affects individuals with mild immunosuppression, such as those with COPD, alcoholism, diabetes, or connective tissue disease^(4,23,36)^. In tomography studies, there are signs of bronchopneumonia and consolidations, predominantly in the upper lobes, which evolve to thick-walled cavitary lesions, including aspergillomas^(4,23)^, as depicted in Figure 7.

**Figure 8:**
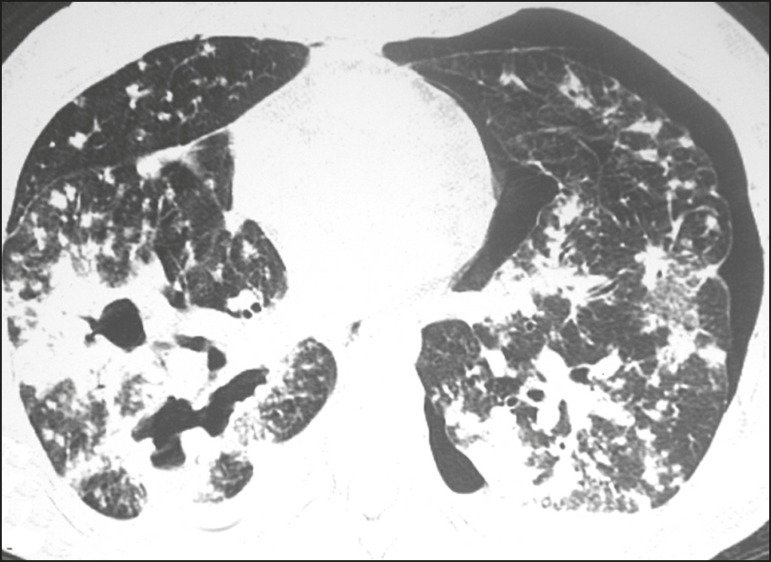
Patient with histoplasmosis. Axial high-resolution computed tomography of the chest, showing centrilobular opacities and bilateral sparse foci of consolidation, in addition to a cavitary lesion in the right lower lobe. Small pneumothorax on the left.

Cavitary lesions can be seen in acute and chronic histoplasmosis (Figure 8), tending to be more common in patients with chronic lung diseases, such as COPD, and individuals with immunosuppression^(36)^. In the chronic cavitary form, the symptoms are similar to those of post-primary tuberculosis, including a low-grade fever, cough, hemoptysis, and weight loss. Imaging studies show reticular opacities accompanied by cavitary lesions, predominantly in the upper lung fields^(2)^.

**Figure 9:**
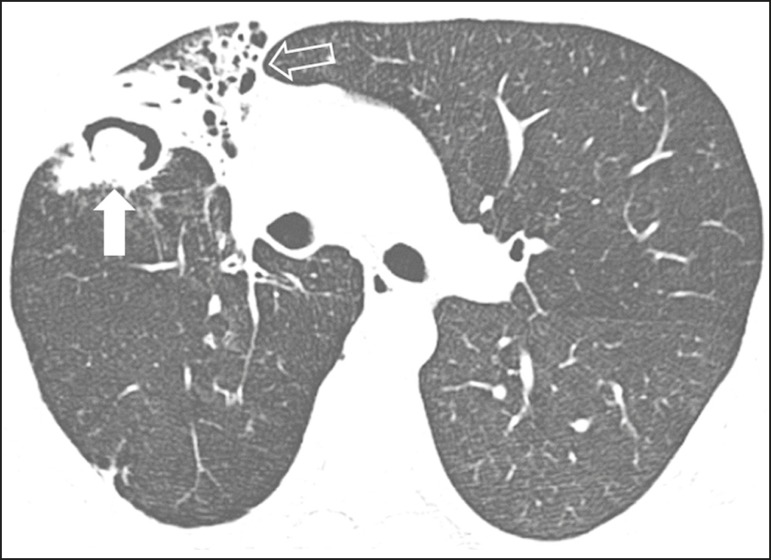
Patient with saprophytic fungal infection. Axial tomography of the chest, with a lung window setting, showing a cavitary lesion in the middle lobe, with a mobile nodule in the interior (aerial crescent), a characteristic aspect of saprophytic aspergillosis (white arrow). As an additional finding, bronchiectasis in the same wolf (leaked arrow).

In the saprophytic form of aspergillosis, the fungus grows inside cavitary lesions, especially in those caused by tuberculosis, sarcoidosis, or other infectious diseases. A nodule or mobile mass within a cavitation, as depicted in Figure 9, is the typical manifestation of saprophytic aspergillosis^(23)^.

### Airway disease

**Figure 10:**
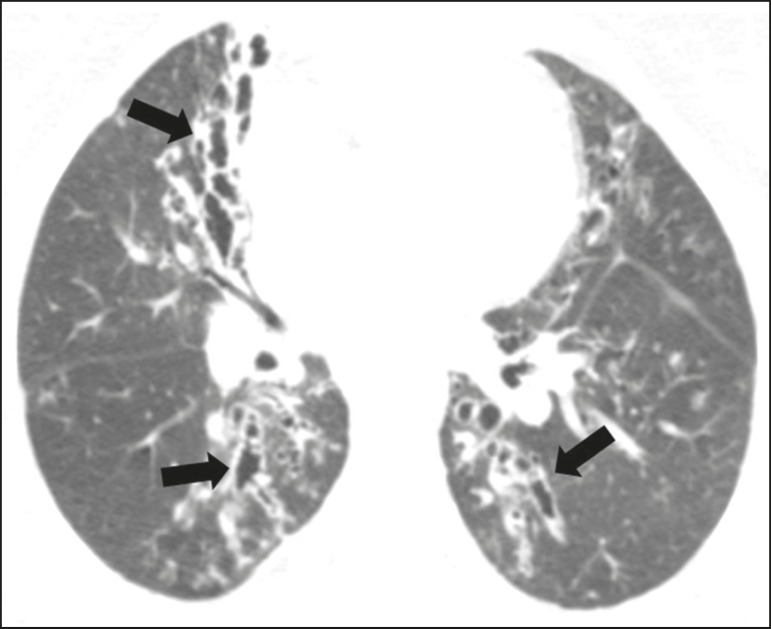
Patient with asthma and allergic bronchopulmonary aspergillosis. Axial tomography of the chest, with a lung window setting, showing predominantly central varicose bronchiectasis accompanied by bronchial parietal thickening (arrows).

Allergic bronchopulmonary aspergillosis is an uncommon clinical manifestation of airway disease and results from a complex hypersensitivity reaction to *Aspergillus* sp. It especially affects individuals with asthma of long duration, cystic fibrosis, or Kartagener syndrome, as well as lung transplant recipients^(4,23)^. Aspergillosis leads to increased mucus production and impaired mucociliary clearance, resulting in damage to the bronchial wall, mucoid impaction, and bronchiectasis^(23)^. The tomographic findings that merit attention are central (segmental and subsegmental) bronchiectasis, especially in the upper lung fields, together with mucoid impaction (Figure 10). It should be noted that the mucoid impaction is hyperdense or contains calcifications in approximately 30% of the patients^(23)^.

**Figure 11:**
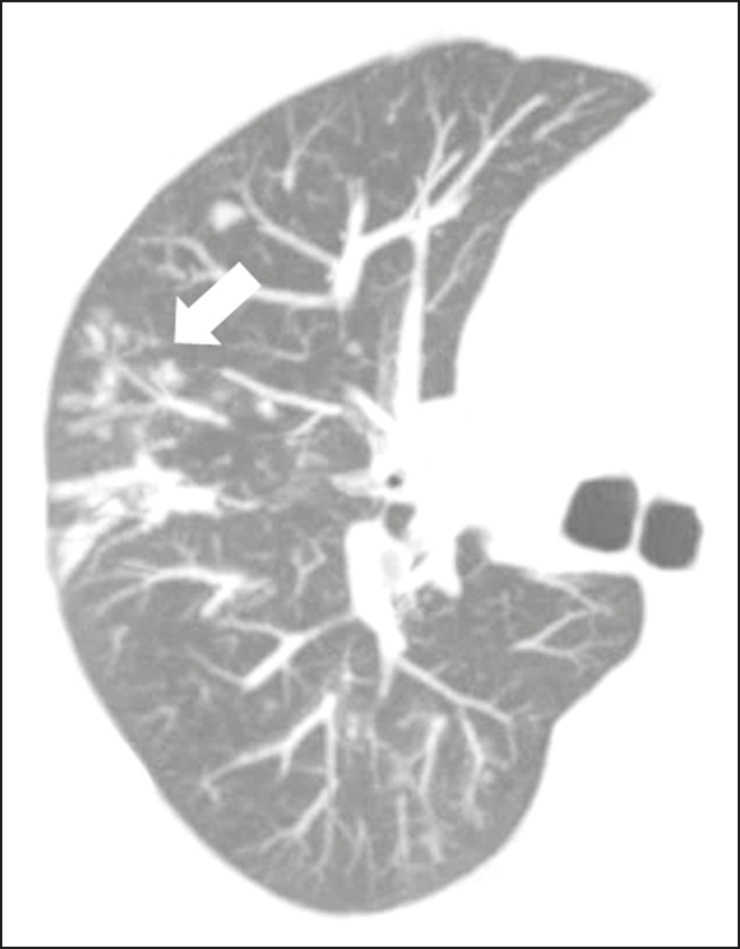
Patient with acute myeloid leukemia and invasive pulmonary aspergillosis. Axial chest tomography with maximum intensity projection reconstruction showing centrilobular opacities grouped in a tree-in-bud arrangement (arrow).

Aspergillosis can manifest as invasive airway disease, especially in immunocompromised individuals with neutropenia and those with AIDS. The disease spectrum extends from the large airways (trachea) to the small airways. Involvement of the small airways can occur in the form of bronchiolitis, with centrilobular nodules, “tree-in-bud” nodules, and peribronchovascular consolidations, or in the form of bronchopneumonia (Figure 11), a pattern similar to that of bronchopneumonia in general^(23)^. Although rare, aspergillosis involvement of the trachea and bronchi (invasive tracheobronchial aspergillosis) can occur in immunocompromised individuals, leading to ulcers or extensive/pseudomembranous necrosis on the mucosal surface^(37)^.

### Mediastinal involvement

**Figure 12:**
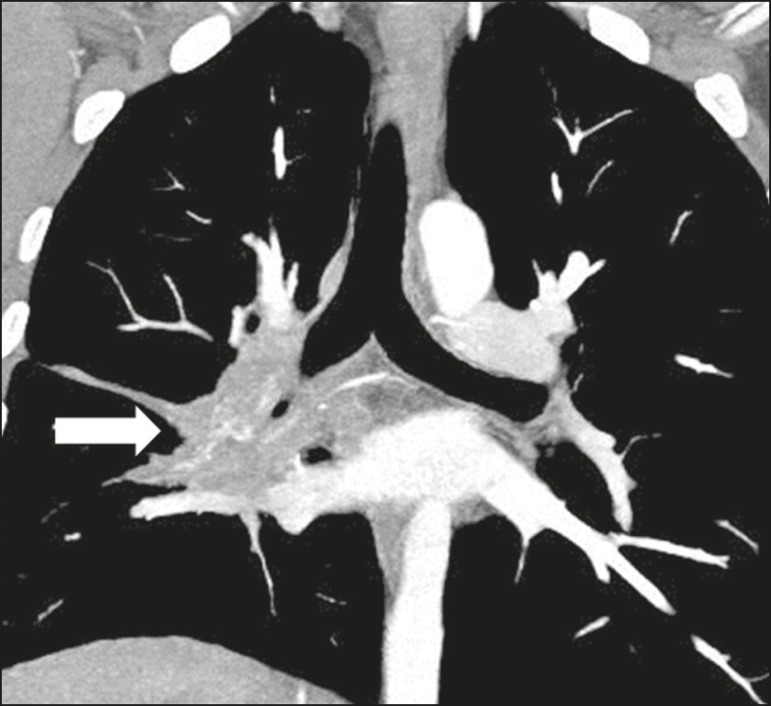
Patient with fibrosing mediastinitis. Contrast-enhanced coronal tomography of the chest, with a mediastinal window setting and maximum intensity projection reconstruction, showing a right-sided subcarinal, hilar infiltrative lesion (arrow), obliterating the pulmonary artery of that side, with an extensive network of collaterals.

Fibrosing mediastinitis is related to the host immune response, which results in proliferation of dense fibrous tissue in the mediastinum^(35)^, although the cause of the immune response is undefined in some cases. It can be idiopathic, secondary to infiltrative neoplastic diseases or infectious diseases, including tuberculosis, aspergillosis, mucormycosis, cryptococcosis, and especially histoplasmosis, the last being a leading cause of the disease, especially in endemic areas^(38)^. It usually affects younger individuals, leading to varied clinical findings, mainly related to compression of vital mediastinal structures. Tomography shows a solid infiltrative lesion, with soft-tissue density and variable enhancement (Figure 12), typically in the middle mediastinum, with or without calcifications.

Imaging plays an important role in depicting and quantifying the involvement of vital, cardiovascular, or airway structures. In the pulmonary compartment, there can be secondary repercussions, such as infarcts or thickening of the interlobular septa, due to obliteration of arterial or venous branches, respectively^(38)^.

Mediastinal lymph node involvement is seen in some mycoses, especially histoplasmosis, cryptococcosis, coccidioidomycosis, and, less commonly, paracoccidioidomycosis^(2-4,20,39)^. Disseminated lymph node enlargement has been described in cryptococcosis and histoplasmosis, especially in immunocompromised individuals. In some cases, necrotic lymph nodes are observed^(4,33)^.

### Pleural effusion

Pleural effusion is uncommon in individuals with pulmonary fungal infections^(4,40)^. Among such infections, the frequency of pleural effusion is slightly higher in mucormycosis^(4)^.

### Chest wall involvement

Thoracic wall involvement can be observed as an extension of the pulmonary infection occurring in aspergillosis, manifesting as lytic lesions in costal arches and vertebrae, being best characterized in tomography and magnetic resonance imaging studies^(41)^. Involvement of the chest wall, in the form of costochondritis, has been reported in intravenous drug users^(42)^.

## CONCLUSION

Several imaging patterns may be observed in pulmonary fungal infections, such patterns often being nonspecific and having the potential to overlap among the various agents, leading to numerous differential diagnoses, including infectious and noninfectious causes. With the objective of systematizing the approach to these diseases, we have reviewed some patterns, including the basic patterns in the pulmonary parenchyma and in extrapulmonary involvement.

Given the multiplicity of presentations of such diseases, there is a need for attending physicians to be familiar with the manifestations. For the correct diagnosis and management of these diseases, it is also necessary that the multidisciplinary team follow patients closely.
